# Neural Correlates of Syntax and Proto-Syntax: Evolutionary Dimension

**DOI:** 10.3389/fpsyg.2018.02415

**Published:** 2018-12-14

**Authors:** Ljiljana Progovac, Natalia Rakhlin, William Angell, Ryan Liddane, Lingfei Tang, Noa Ofen

**Affiliations:** ^1^Linguistics Program, Wayne State University, Detroit, MI, United States; ^2^Department of English, Wayne State University, Detroit, MI, United States; ^3^Lifespan Cognitive Neuroscience Program, Institute of Gerontology, Wayne State University, Detroit, MI, United States; ^4^Department of Psychology, Wayne State University, Detroit, MI, United States

**Keywords:** syntactic processing, evolution of syntax, proto-syntactic “fossils”, functional MRI, Broca’s area, basal ganglia

## Abstract

The present fMRI study tested predictions of the evolution-of-syntax framework which analyzes certain structures as remnants (“fossils”) of a non-hierarchical (non-recursive) proto-syntactic stage in the evolution of language (Progovac, 2015, 2016). We hypothesized that processing of these structures, in comparison to more modern hierarchical structures, will show less activation in the brain regions that are part of the syntactic network, including Broca’s area (BA 44 and 45) and the basal ganglia, i.e., the network bolstered in the line of descent of humans through genetic mutations that contributed to present-day dense neuronal connectivity among these regions. Fourteen healthy native English-speaking adults viewed written stimuli consisting of: (1) full sentences (FullS; e.g., *The case is closed*); (2) Small Clauses (SC; e.g., *Case closed*); (3) Complex hierarchical compounds (e.g., *joy-killer*); and (4) Simple flat compounds (e.g., *kill-joy*). SC (compared to FullS) resulted in reduced activation in the left BA 44 and right basal ganglia. Simple (relative to complex) compounds resulted in increased activation in the inferior temporal gyrus and the fusiform gyrus (BA 37/19), areas implicated in visual and semantic processing. We discuss our findings in the context of current theories regarding the co-evolution of language and the brain.

## Introduction

It has been suggested that the study of how syntactic structures are represented and processed in the brain has reached an impasse, failing to achieve cross-fertilization between the fields of Linguistics and Neuroscience (e.g., Poeppel and Embick, 2005; also Fedorenko and Kanwisher, 2009). The reason for the difficulty of using the insights about the nature of syntactic representations and computation to inform our knowledge about functional organization of the brain and vice versa was said to lie in the inherent mismatch in conceptual granularity as well as ontological incompatibility between the concrete biological units of neuroscience and the abstract syntactic postulates (Poeppel and Embick, 2005). We propose that the considerations of the evolution of syntax, as outlined in Progovac (2015, 2016), provide a way of bringing together the postulates from Theoretical Syntax and Cognitive Neuroscience (see also Progovac et al., 2018). This framework, by contrasting modern structures (syntax) with ancestral structures (proto-syntax), introduces linguistic constructs of the granularity commensurate with the tools used in neuroscience to probe the neural correlates of syntactic computation. We argue that this approach opens a new avenue for neurolinguistic research with a potential to provide the necessary points of contact with other relevant fields.

The literature linking neuroscience and language evolution largely centers around the question of how language first emerged in the descent of humans. More precisely, the issues often debated in the literature include whether or not language processing can be grounded in neurobiological structures and cognitive functions found in non-human primates, what form the earliest protolanguage had, and what changes in the brain/cognition led to the transition from the protolanguage to language (Bickerton, 1995; Wray, 1998; Arbib, 2012, 2016; Bornkessel-Schlesewsky et al., 2015). For example, Arbib (2012, 2016) argued that language emerged as a result of biological and cultural co-evolution, originating from the brain structures and functions allowing for imitation and pantomime (mirror neurons), as well as social and cognitive capacities (intention reading and symbolic thinking). Under this hypothesis, protolanguage was a system of proto-sign, holophrastic in nature. Under this theory, the initial combinatorial sign language that emerged from the holophrastic stage eventually gave rise to language in the auditory-articulatory modality. Under an alternative, “compositional” view, protolanguage (in the species predating Homo Sapiens) consisted of words that could be combined without syntactic structure, which evolved into language by adding syntax (Bickerton, 1995).

Our present study focuses on the initial stages of language evolution at the point at which combinatorics (simple syntax) just started emerging, prior to the arrival at the hierarchical and recursive syntax. Thus, we adopt a gradualist view of the evolution of syntax, which is more compatible with the compositional view of protolanguage, and which identifies clear communicative benefits of combining even the crudest of vocabulary items (Progovac, 2016). Our study also addresses the issue of recursion, brought up by Hauser et al. (2002), and later discussed in many contributions, including, e.g., Berwick and Chomsky (2011). Theirs is a saltationist view of the evolution of syntax, claiming that syntax is an all-or-nothing package, an undecomposable block, which could only evolve at once in its full complexity, and in its hierarchical/recursive form. However, in contrast to this saltationist view of the evolution of syntax, and more in line with Jackendoff and Pinker’s (2005) view, the idea behind our proposal is that there was an initial simple (but coherent) stage of syntax which was not recursive, and we show that modern-day approximations of this syntax (“fossils”) still exhibit resistance to recursion in the sense that they cannot embed (an elaborate discussion of this issue can be found in Progovac, 2015, 2016). We address the recursion controversy in a very specific, tangible manner, by identifying non-recursive syntactic “fossils” and by contrasting their processing to the processing of their hierarchical/recursive counterparts.

As pointed out by a reviewer, the gradualist approach we advocate here is compatible with the view of evolutionary continuity between humans and non-human primates, including the view that envisions a gestural beginning of language with a gradual transition to vocal speech. According to this view, great apes’ manual gestures are homologous to language (Corballis, 2010; Arbib, 2012) in that they are intentional, communicative behaviors, rooted in manual praxis and subject to social learning (Pollick and De Waal, 2007; Arbib et al., 2008).

Our rationale for the relevance of evolutionary considerations to neurolinguistic investigations is consistent with the idea (Pinker and Bloom, 1990; Jackendoff, 1999, 2002; Deacon, 2003; Progovac, 2010, 2015) that language systems in the human brain evolved gradually, representing co-evolution of brain and language. In other words, the brain changed via natural selection partly due to pressures to process increasingly more complex linguistic structures, e.g., newly added layers of hierarchical syntax. Under this theory, even the structures typically considered basic in syntactic analysis are decomposable into evolutionary primitives, with remnants (“fossils”) of ancestral structures still co-existing alongside (or within) modern syntactic structures. Furthermore, it may be possible to isolate certain brain networks that are specialized for processing structures of different degrees of syntactic elaboration, as reflected in the stages of language evolution. The rationale behind this proposal is that complex, uniquely human, grammatical patterns require more support by the most recently evolved/enhanced neural networks than do their flatter proto-syntactic counterparts, which in turn may show a less streamlined and more diffuse distribution across the brain, as well as more individual variability (Progovac et al., 2018).

Thus, our main hypothesis is that processing of less hierarchical (proto-syntactic) structures would produce reduced activation in the more recently enhanced brain networks associated with syntactic processing. We rely on the neurobiological theory of syntactic processing that posits a cortical-subcortical network, which includes (among others) the Broca’s area, in particular BA 44, a key area anchoring the processing of syntactic hierarchies (Friederici, 2017; see also Opitz and Friederici, 2007), and basal ganglia (e.g., the striatum), which integrates cortical inputs during syntactic computation (Teichmann et al., 2009, 2015; Szalisznyó et al., 2017). The view that Broca’s area is not the sole center for syntactic processing, but rather is part of a larger circuit that involves subcortical structures has been accumulating support (Gibson, 1996; Lieberman, 2000, 2009; Vargha-Khadem et al., 2005; Ardila et al., 2016a,b; Ullman, 2006). Our hypothesis directly engages the discovery that the connectivity of the Broca’s–Basal Ganglia network was strengthened recently in evolution, in the line of descent of humans (e.g., Enard et al., 2009; Hillert, 2014; Dediu, 2015).

We contend that discovering evolutionary aspects of the neurosyntactic architecture by decomposing syntax into its evolutionary components and tracing differential brain activation during processing of ancestral and modern structures would inform both Linguistics and Cognitive Neuroscience and lead to breakthroughs in identifying components of syntactic processing jointly validated through syntactic and neuroimaging research.

### Evolutionary Proposal in Brief

In generative syntax (e.g., Chomsky, 1995; Adger, 2003), modern sentences are analyzed as hierarchical structures, consisting of several layers composed in a binary fashion. The following is the *partial* hierarchy of layers involved in the construction of a typical sentence:

(1) TP > vP > SC/VP

Here TP is a Tense Phrase layer (sentence/clause), vP a transitive (higher) verb Phrase, VP the basic (intransitive) Verb Phrase, and SC a Small Clause. Syntactic derivation of a basic transitive sentence, such as *Elena will grow tomatoes*, progresses from the most basic, inner layer, the SC/VP *grow tomatoes*, where the syntactic function of the noun phrase (NP) “tomatoes” is not yet determined as either the subject or the object. Once the transitivity layer (vP) is added, it enables grammaticalized differentiation between subjects and objects. The TP layer, in this case headed by *will*, is then superimposed over the verbal layers allowing for verb finiteness and structural phenomena associated with it. Sentences and phrases in this framework can exhibit even more layers of structure, resulting in highly hierarchical constructs.

English examples (2) and (3) demonstrate how transitive (2) and intransitive (3) sentences are derived, and how the boundary between them, as well as between subjecthood and objecthood, can get blurred.

(2) Elena will grow tomatoes.
  (a) [_SC/VP_] grow tomatoes] →
  (b) [_vP_ Elena [_SC/VP_ grow tomatoes]] →
  (c) [_TP_: Elena will [_vP_ Elena [_SC/VP_ grow tomatoes]]]

(3) Tomatoes will grow. / Elena will grow.
  (a) [_SC/VP_ grow tomatoes/Elena] →
  (b) [_TP_: Tomatoes/Elena will [_SC/VP_] grow tomatoes/Elena]]

The cross-out notation marks the underlying position in which the subject was initially merged, prior to moving to TP. In this theory, movement of the subject to TP is an automatic reflex of TP layering and abstract feature checking associated with TP (i.e., a purely syntactic phenomenon not associated with semantic considerations). Importantly, the small clause/VP layer provides the foundation for building both intransitive and transitive structures and for superimposing both vP and TP layers. The small clause analysis is at the heart of this syntactic framework and dates back to the proposals in, e.g., Stowell (1983), Kitagawa (1985, 1986), and Koopman and Sportiche (1991). Thus, this analysis has withstood the test of time and empirical scrutiny, being one of the most stable postulates in this theoretical framework.

The evolutionary proposal in Progovac (2015, 2016) relies only on such stable postulates with a clear empirical basis. It uses the theoretically based hierarchy in (1) to offer a precise method of reconstructing evolutionary stages of syntax, as formalized in (4) from Progovac (2015:7):

(4) “Structure X is considered to be evolutionarily primary relative to Structure Y if X can be composed independently of Y, but Y can only be built upon the foundation of X.”

This approach allows for the small clause layer to be reconstructed as the initial evolutionary stage of grammar and suggests that building a modern sentence in some sense retraces the evolutionary steps. Progovac’s (2015: 3) reconstruction arrives at a proto-grammar characterized as a “flat, tenseless, intransitive, two-slot mold with one verb-like and one noun-like element, in which the subject/object distinction could not be expressed grammatically.” Imposing additional layers of structure (such as TP or vP) upon the foundational SC necessarily yields a hierarchical, layered construct.

Importantly, one finds approximations of the ancestral structures (or “living fossils” in the sense of Jackendoff, 1999, 2002) in modern languages (but see Miyagawa, 2017 for an opposing view)^1^. One example would be verb-noun compounds, such as *pick-pocket*, *kill-joy*, *tattle-tale*, *cry-baby*, and *rattle-snake^2^*, which do not feature vP and TP layers. These are “essentially SCs created by two-slot grammars with one verb and one noun, without a possibility for any elaboration or for distinguishing subjects from objects” grammatically (Progovac, 2016: 3).

In contrast, more elaborated –*er* compounds (e.g., *tax-payer, risk-taker, trouble-maker, and heart-breaker*) are more predictable in their meaning: the noun inside these compounds is necessarily object-like (contrast *table-turner*, i.e., somebody who turns tables, to *turn-table*, i.e., table that turns; gramophone). They can be analyzed as built upon the foundation of the simple counterparts, with the subject-like –*er* piece added to the verb-noun foundation, as shown in (5) (see Progovac, 2015 for detailed discussion). Thus, these compounds involve an overlay of abstract morpho-syntactic structure and exhibit morphological productivity in English, in the sense that one can freely create new ones, including the ones never heard before (e.g., *pumpkin-smasher*; *chalk-eater*)^3^.

(5) [_SC_ kill joy] → [–er [_SC_ kill–joy]] → [[joy-kill] –er]

Another type of “fossil” is illustrated in (6–6′). These are structures analyzed by Progovac as SCs without a TP (Tense Phrase) layer. The paucity of abstract syntactic structure is made obvious when they are considered in contrast to the full TP counterparts (7–7′)^4^.

(5) Case closed. Mission accomplished. Problem solved. Crisis averted.
(6′) [_SC_ case closed]
(7) The case is closed. The mission was accomplished. The problem is solved.
(7′) [_TP_ [_DP_ the case] is [_SC_ (the) case closed]]

Of note is the abstract nature of modern syntactic functional categories that distinguish the clauses above: determiners (in particular articles *a*, *the*) and auxiliary verbs (e.g., *is*, *was*), far from being concrete and imageable, contribute a great deal to the abstractness of modern layered syntax. Also worth mentioning is that examples like (6) are not just elliptical versions of (7), as they show distinct syntactic behavior (see Progovac, 2013 for a review article on this topic). To take just one example, SCs cannot embed (8), the way their TP counterparts can (9), and thus are not recursive:

(8) ^∗^I believe (that) [crisis averted]. (‘^∗^’ marks ungrammatical examples)
(9) I believe (that) [the crisis was averted].

Thus, in addition to the other fossil characteristics, SCs investigated in this study also exhibit a lack of recursion, suggesting that recursion goes hand in hand with layered syntax, but that coherent simpler syntax is still possible without either (a detailed discussion of this matter, based on crosslinguistic evidence, can be found in Progovac, 2015).

### Previous Neuroimaging Research

Recent findings are converging on the conclusion that language processing involves a distributed network of interconnected modules in the left hemisphere, with the right hemisphere also being involved (see, e.g., Embick et al., 2000; Friederici et al., 2000; Moro et al., 2001; Bookheimer, 2002; Brennan et al., 2012). Furthermore, various findings suggest that syntax is not a monolith, but a complex phenomenon that recruits multiple loci in the brain. Thus, Grodzinsky and Friederici (2006) conclude that each subpart of the linguistic system, including syntax, is neurologically decomposable into subsystems with a distinct neuro-functional architecture. These findings are consistent with the gradualist approach to the evolution of syntax we advocate.

For example, sentences with constituents moved from their underlying positions have been reported to exhibit increased activation in the left Inferior Frontal Gyrus (IFG), clustering around (and outside) Broca’s area: Brodmann Areas (BA) 44, 45, 46 and 47 (Stromswold et al., 1996; Ben-Shachar et al., 2004; Constable et al., 2004; Friederici et al., 2006; Grodzinsky and Friederici, 2006; Grodzinsky, 2010), as syntactic movement operations arguably require more syntactic space/layering to be executed than simpler syntactic structures with canonical word order. Others (Rogalsky and Hickok, 2011; Santi and Grodzinsky, 2012; Matchin et al., 2014; Santi et al., 2015) argued that the Broca’s and basal ganglia networks are relevant for various types of linguistic processing, including phonological (see, e.g., Heim et al., 2009), in addition to processing hierarchical syntax, which, we contend, was one of the key drivers of the evolution of these networks^5^. For further findings correlating an increase in syntactic complexity to an increase in neural activation in certain specific areas of the brain, the reader is referred to Just et al. (1996), Caplan (2001), Indefrey et al. (2001), Pallier et al. (2011), and Brennan et al. (2012). In an fMRI experiment, Progovac et al. (2018) found that the basal ganglia showed increased activation, both on the left and on the right, with the processing of Serbian transitive sentences (instantiating a vP layer), in contrast to matched “middle” sentences (analyzed as lacking the vP layer)^6^.

Building on the previous studies, and equipped with the evolutionary method of syntactic reconstruction, we tested hypotheses regarding the processing of various structural layers of syntax. Specifically, we investigated whether proto-syntactic structures (e.g., SCs and flat compounds) are processed differently from their more complex hierarchical counterparts, in the hope of isolating neural correlates of these distinctions.

## Study Goals and Hypotheses

The goal of the present study was to investigate patterns of brain activation during on-line sentence processing of two types of syntactic structures: flat and hierarchical. We hypothesized that these two types of structures would be associated with differential patterns of brain activation, namely: (1) processing of more hierarchical structures would be associated with greater activation in the brain areas and networks known to specialize for language/syntax (i.e., left-lateralized Broca’s area, and the basal ganglia); (2) processing of flatter structures (proto-syntax) would result in greater activation in areas outside of these specialized language networks. We focus on these contrasts in English, but this method can be applied to a variety of language types, with different languages providing different testing opportunities (e.g., Progovac et al., 2018).

## Methods

### Participants

Fourteen healthy adults (6 Female, age range 21–52, mean = 28.4, *SD* = 11.1) participated in this study. As determined in a self-report pre-scan screening, participants had no language impairments and they were not previously diagnosed with major psychiatric or neurological disorder. Right handed (Edinburgh Handedness Inventory, Oldfield, 1971) native monolingual speakers of English were included. Written informed consent was obtained from all participants.

### Stimuli

The experiment contained five conditions: SC (10), FullS (11), Two-word Control Sentences (2WordS) (12), Simple Flat Compounds (13), and Complex Hierarchical Compounds (14). We included 2WordS to control for the potentially confounding effect of the difference in the number of words between the FullS and SC conditions. 2WordS consisted of FullS that matched the length (two words) of the SC stimuli without missing the TP layer of the FullS.

(10) Crisis averted. Point taken. Lesson learned.
(11) The crisis was averted. The point is taken. The lesson was learned.
(12) Disruption occurred. Fires spread. Fog lifted. Love stinks.
(13) pick-pocket, scare-crow, turn-coat, hunch-back, wag-tail, kill-joy
(14) joy-killer, woman-hater, boot-licker, risk-taker, truth-seeker, ball-breaker

We predicted increased activation in the areas of the brain that are part of the known specialized language network (i.e., Broca’s area and the basal ganglia) during the processing of FullS and 2WordS compared to SC, and Complex Compounds compared to Simple Compounds. We also expected that the flatter structures (SC and Simple Compounds) will be associated with a more diffuse pattern of activation, as such stimuli are expected to rely on more general cognitive processing strategies that may have been in place before complex human language emerged and contributed to the modification of the brain (Progovac et al., 2018: 7; see also Ansaldo et al., 2015).

To control for the effect of increasing semantic complexity with increased syntactic complexity, we kept semantic meaning of the FullS and SC pairs constant by constructing these stimuli using the same content words in the FullS and corresponding SCs, while allowing the two sentence types to have different degrees of syntactic elaboration. This is commonly accomplished in the literature by using pseudowords in lieu of real content words, while retaining functional elements and measuring brain response to syntactic violations (e.g., Moro et al., 2001). Our method allowed us to look at processing of real words (rather than pseudowords) in real time and still to be able to isolate syntactic phenomena.

Unfortunately, the two types of compounds could not be similarly matched, as these two conditions did not allow for constructing pairs with the same content words (except in the case of *kill-joy/joy-killer*), due to Simple Compounds being rare and no longer productive in English^7^. We matched the two types of compounds on frequency, using the Corpus of Contemporary American English (COCA), which consists of 533,788,932 words (Davies, 2008). The mean frequency for the simple compounds was 256.75 (*SD* = 457.27), and for the complex compounds 334.7 (*SD* = 915.89). Results of a 2-tailed *t*-test indicated that this difference was not significant (*p* = 0.71).

### Procedure

**FIGURE 1:**
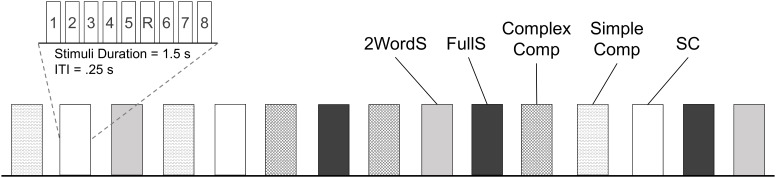
Schematic representation of the experimental design. Functional activations associated with the processing of stimuli from 5 experimental conditions (SC, Small Clauses; FullS, Full Sentences; 2WordS, Two-word Sentences; Simple Comp, Simple Compounds; Complex Comp, Complex Compounds) were tested using a block design. In each block 8 unique stimuli were presented and participants were instructed to read the text and indicate with a button press any stimuli repetition (in this example, stimuli number 5 denoted by R). Each block lasted 15.75 s and was followed by a 10 s inter-block interval. Three blocks were presented per each of the experimental conditions for a total of 24 stimuli per condition. The total run time was 6 min 26 s. ITI, interval between stimulus presentations within a block; Block and Stimuli within a block are represented by vertical box.

A total of 120 stimuli, 24 per condition, were presented visually as centered white text on a black background (Times New Roman, 80-point font) (see Appendix for complete list of stimuli). The 24 stimuli from each of the 5 conditions were presented in 3 blocks, 8 unique stimuli from a single condition in each block. There were 3 blocks per each of the 5 conditions for a total of 15 blocks altogether. Each stimulus was presented for 1500 ms followed by a 250 ms during which a fixation crosshair was presented in the middle of the screen. To ensure that participants adequately engaged with the stimuli, we embedded a simple repetition detection task (1-back) in each block; one of the stimuli was presented twice in succession and participants were asked to indicate with a button press when such repetition occurred. Each block lasted a total of 15.75 s, and was followed by 10 s inter-block-interval, during which a fixation screen was presented. Two pseudorandom orders for the presentation of blocks of different experimental conditions were used, each assigned to about half of the participants. Orders were generated with one restriction that no single condition was repeated in two successive blocks (see Figure 1). PsychToolbox in MATLAB was used for the presentation of the stimuli and recording of responses. As behavioral measures, we calculated the accuracy and reaction times per response to the repeated stimuli in each block.

### MRI Data Acquisition

MRI data were acquired at the Wayne State University MR Research Facility using a 3T Siemens Verio scanner. Whole-brain T1-weighted structural images were acquired using an MPRAGE sequence (176 coronal slices; repetition time (TR) = 1680 ms, echo time (TE) = 3.51 ms, flip angle = 9°, field of view = 256 mm, 176 × 256 voxels, voxel size = 0.7 mm × 0.7 mm × 1.3 mm). Whole-brain T2^∗^-weighted multiband accelerated EPI pulse sequence functional images were acquired during the time participants completed the experimental tasks in a single run (75 slices parallel to the AC-PC plane; TR = 2000 ms, TE = 30 ms, flip angle = 90° voxel size = 2 mm × 2 mm × 2 mm, multiband factor = 3, duration = 6 min 26 s, total 197 volumes).

### MRI Data Analyses

Functional data were analyzed using SPM8 package (Wellcome Department of Imaging Neuroscience, London, United Kingdom). Preprocessing included standard processes for motion correction, normalization to template (Montreal Neurological Institute, MNI), and smoothing with a 5-mm full-width half-maximum Gaussian kernel. Statistical analyses of fMRI data were conducted using general linear modeling (GLM), as implemented in SPM8. First-level analyses included the 5 experimental conditions modeled with separate regressors: SC, FullS, 2WordS, Simple Compounds, and Complex Compounds. The BOLD response was modeled by convolving a canonical hemodynamic response (HDR) function with a boxcar function spanning the duration of the block (15.75 s) and temporal derivatives of each block were included in the GLM to account for temporal shifts in the response of the stimuli (Friston et al., 1998). Specific contrasts of interest were computed for each individual and combined into whole brain group-level analyses. These contrasts included: (i) FullS versus SC; (ii) 2WordS versus SC; and (iii) Simple Compounds versus Complex Compounds. The thresholds for all three contrasts were set at *p* < 0.005, with strict extent threshold of 100 contiguous voxels, minimizing the possibility of the findings being false positive. To identify the common regions for (i) and (ii), we conducted a conjunction analysis. By using these contrasts at the set voxel threshold of *p* < 0.005, the conjunction analysis provides an effective threshold of *p* < 0.000025. For completeness and given the stringent effective threshold applied in reporting the results of the conjunction analysis, we included reported results in the tables with a relaxed extent threshold of 9 contiguous voxels in the resulting conjunction map.

In a complementary analysis, we assessed syntax-related activation in *a priori* anatomically defined regions of interest (ROIs). Six ROIs were generated, according to the same approach we used in the past (Progovac et al., 2018), using the Wake Forest University Pickatlas tool spanning the left and right Brodmann Area (BA 44, BA 45), and basal ganglia (combined caudate and putamen). Parameter estimates values per condition were extracted from these ROIs. Repeated measures ANOVAs and planned comparisons were conducted with the mean extracted contrast value from each of the six ROIs described above. Reported findings were significant at *p* < 0.05. We first tested significant main effects of condition in a repeated measures ANOVA including three conditions (SC, FullS, and 2WordS), and follow-up significant main effects with paired *t*-tests (2-tailed). In addition, within these anatomically defined ROIs we tested for potential difference between Simple and Complex Compounds using a paired *t*-test (2-tailed).

## Results

### Behavioral Data

Overall, participants responded accurately to repeated stimuli (*Mean* = 0.99, *SD* = 0.03). Accuracy rate did not differ between conditions, *F*(4,48) = 0.74, *p* = 0.57 (SC: *Mean* = 0.97, *SD* = 0.09; FullS: *Mean* = 1.00, *SD* = 0.00; 2WordS: *Mean* = 1.00, *SD* = 0.00; Simple Compounds: *Mean* = 1.00, *SD* = 0.00; Complex Compounds: *Mean* = 0.97, *SD* = 0.09). In terms of reaction time, there was marginal difference by condition (*F*(4,48) = 3.27, *p* = 0.05). Reaction time for the FullS condition (*Mean* = 0.65, *SD* = 0.18) was longer than for the three other conditions (SC: *Mean* = 0.58, *SD* = 0.15; 2WordS: *Mean* = 0.60, *SD* = 0.14; Complex Compounds: *Mean* = 0.59, *SD* = 0.14; *p*s < 0.003), but not significantly different from Simple Compounds condition (*Mean* = 0.58, *SD* = 0.10, *p* = 0.07).

### Reduced Brain Activation for Small Clauses in Syntax-Related Regions

**FIGURE 2:**
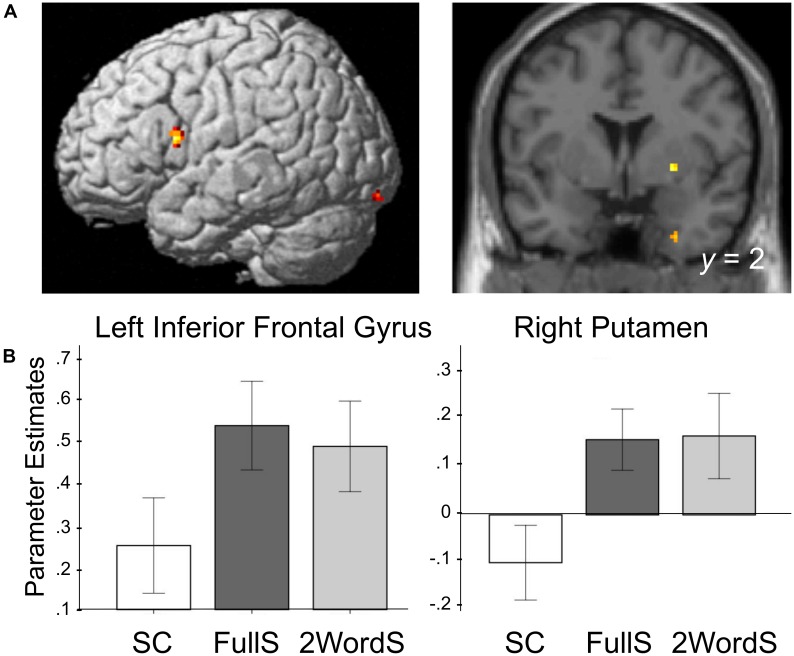
Processing of Small Clauses is associated with reduced activation in the left IFG (BA 44) and the right putamen. **(A)** Activation maps are rendered on a brain template depicting brain regions in which activation for Small Clauses was lower compared to activation for Full Sentences and for Two-word Sentences. These brain regions include regions in the Inferior Frontal Gyrus (*x* = –48, *y* = 8, and *z* = 16) and the Putamen (*x* = 26, *y* = 2, and *z* = –2). Conjunction analysis was made using contrast maps of Small Clauses compared to Full Sentences, and Small Clauses compared to Two-word Sentences, each threshold at *p* < 0.005 (conjunctive *p* < 0.000025). Clusters identified by the conjunction composed of at least 9 contiguous voxels are reported. **(B)** Mean parameter estimates per condition extracted from the functionally defined regions of interest identified in the conjunction analysis shown in **A**. SC, Small Clauses; FullS, Full Sentences; 2WordS, Two-word Sentences.

To identify regions that showed differences in the activation for the SC condition, we created group-level contrasts comparing activation for FullS versus SC as well as for 2WordS versus SC conditions. Brain regions in the occipital and temporal lobe, left inferior frontal gyrus (BA 44, Broca’s area) and right putamen (basal ganglia) showed less activation to SC compared to FullS (Table 1A). Also, left inferior frontal gyrus and posterior cingulate gyrus showed less activation to SC compared to 2WordS (Table 1B). Critically, we conducted a conjunction analysis between these two contrasts assessing the patterns by which SC may differ from these two conditions. Activations in the left inferior frontal gyrus (BA 44, Broca’s area), right putamen (basal ganglia), and in regions in the right temporal and occipital lobes were lower for SC condition compared to both FullS and 2WordS conditions (Table 1C and Figure 2A). For visualization of these effects, parameter estimates were extracted from the clusters in the Broca’s area and the right basal ganglia, as identified by the conjunction analysis. The mean group parameter estimates are depicted in Figure 2B, demonstrating the reduced activation in SC condition compared to both FullS and 2WordS conditions both in Broca’s area and the right basal ganglia (*p*s < 0.001).

**Table 1:** Brain regions where activation for small clauses (sc) differs from full sentences and two-word sentences.

Regions	BA	MNI Coordinates			*t-*Values		#Voxels
				
		*x*	*y*	*z*		
***(A) Full Sentences > SC***						
Right Lingual Gyrus	17	8	-64	0	10.53		10945
Cuneus	18	18	-96	22	9.78	
Left Posterior Cingulate Gyrus	30	-22	-58	6	6.32	
Right Cingulate Gyrus	32	0	30	28	6.56		141
Right/Left Medial Frontal Gyrus	6/8	6	38	32	4.77	
Right Thalamus	NA	24	-28	8	6.44		123
Left Middle Temporal Gyrus	21	-62	-50	-2	6.40		367
Left Inferior Temporal Gyrus	20	-56	-56	-10	6.05	
Left Superior Temporal Gyrus	39	-46	-48	8	5.29	
Left Inferior Frontal Gyrus	9	-50	16	28	6.27		288
Left Inferior Frontal Gyrus	44	-48	8	16	5.14	
Left Precentral Gyrus	6	-32	6	26	5.11	
Right Superior Temporal Gyrus	22	62	-34	8	6.15		227
Right Middle Temporal Gyrus	21	66	-50	-2	5.92	
Right Putamen	NA	24	6	-10	5.88		184
Right Middle Frontal Gyrus	6	28	6	54	5.83		220
Right Precentral Gyrus	6	48	4	54	4.83	
Left Precentral Gyrus	6	-46	0	46	5.65		224
Left Precentral Gyrus	4	-42	-12	60	4.91	
Left Inferior Parietal Lobule	40	-42	-40	46	5.47		116
Left Middle Frontal Gyrus	6	-20	-4	50	5.37		244
Right Insula	13	42	14	2	4.64		120
Right Precentral Gyrus	44	56	16	4	4.19	
***(B) Two-word Sentences > SC***							
Left Inferior Frontal Gyrus	44	-48	8	16		6.25	102
Left Precentral Gyrus	6	-52	-2	6		3.48	
Left Posterior Cingulate Gyrus	30	-24	-58	8		5.38	108
***(C) Conjunction: (A) Full Sentences > SC ∩ (B) Two-word Sentences > SC***					
					***(A) FullS >SC***	***(B) 2WordS >SC***	
Left Inferior Frontal Gyrus	44	-48	8	16	5.14	6.25	40^∗^
Right Putamen	NA	26	2	-2	5.25	4.04	10
Right Putamen	NA	24	6	-10	5.88	3.36	9
Right Uncus	28	28	4	-36	4.50	4.38	11
Right Middle Temporal Gyrus	22	60	-44	0	4.46	3.11	11
Left Posterior Cingulate Gyrus	30	-22	-58	6	6.32	3.36	16^∗^
Left Inferior Occipital Gyrus	18	-26	-96	-16	4.12	4.08	11
Right Middle Occipital Gyrus	18	32	-90	2	5.95	3.01	22
Right Precuneus	7	24	-68	42	6.67	3.75	11
Right Cuneus	19	26	-84	38	4.84	3.05	10


***(A,B)** Clusters identified by contrasting Full Sentences > Small Clauses **(A)** and Two-word Sentences > Small Clauses **(B)**. Voxel-level threshold *p* < 0.005, extent threshold of 100 contiguous voxels. **(C)** Clusters identified by a conjunction analysis between Full Sentence > Small Clauses and Two-word Sentences > Small Clauses (each contrast at a *p* < 0.005 threshold). Conjunction clusters exceeding 9 contiguous voxels are listed. Peak voxel coordinates, mean cluster *t*-values per contrast and number of voxels are reported. ^∗^denotes regions identified in the conjunction analysis clusters that survived the threshold of *p* < 0.005 with 100 contiguous voxels at the base contrasts.*

### Differential Brain Activation for Complex (Hierarchical) vs. Simple (Flat) Compounds

**FIGURE 3:**
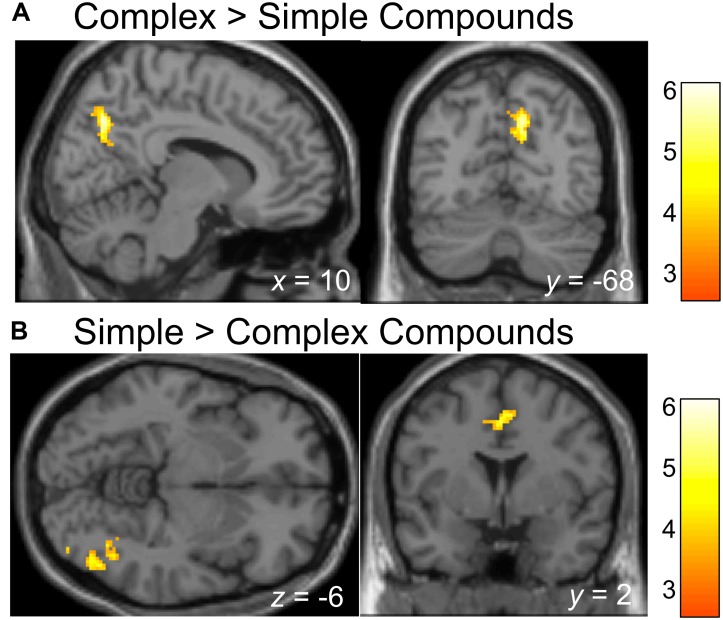
Brain regions showing activation difference when processing Simple compared to Complex compounds. Activation maps are rendered on a brain template depicting regions showing higher activation during the processing of Complex compared to Simple Compounds **(A)**, or higher activation during the processing of Simple compared to Complex Compounds **(B)**. The threshold for depicting effects in activation map was set at *p* < 0.001 with 100 contiguous voxels.

We also investigated whether processing of Complex relative to Simple Compounds involves typical syntax-related regions, and whether other regions demonstrate stronger involvement in processing of Simple relative to Complex Compounds. Whole-brain analyses showed that activation in the right precuneus (BA 7) was higher for the Complex compared to the Simple Compounds (*p* < 0.005; see Table 2 and Figure 3A); however, we did not identify regions associated with typical syntax processing. When examining the opposite contrast, we identified activation in two clusters, one extending through a portion of the inferior temporal gyrus and the fusiform gyrus (BA 37/19), and the second located in the cingulate gyrus (BA24) where activation was higher for Simple compared to Complex Compounds (*p* < 0.005; see Table 2 and Figure 3B).

**Table 2:** Regions showing difference in activation for Complex Compounds compared to Simple Compounds.

Regions	BA	MNI Coordinates			*t*-Values	#Voxels
				
		*x*	*y*	*z*		
***Complex Compounds > Simple Compounds***					
Right Precuneus	7	10	-68	38	5.46	191
	31	18	-62	32	4.76	
		8	-62	26	4.43	
***Simple Compounds > Complex Compounds***					
Right Inferior Temporal Gyrus	37	42	-64	-2	5.44	364
Right Inferior Occipital Gyrus	19	40	-82	-10	5.06	
Right Fusiform Gyrus	37/19	34	-56	-10	4.62	
Left Cingulate Gyrus	24	-2	4	44	4.93	117
Right Cingulate Gyrus	24	2	-4	40	4.53	
Right Medial Frontal Gyrus	6	6	2	50	4.37	


*Clusters identified by analyses comparing activation between Simple and Complex Compounds (clusters consisting of > 100 contiguous voxels at a threshold of *p* < 0.005).*

### Reduced Activation for Small Clause in Anatomically Defined Syntax-Related ROI

**FIGURE 4:**
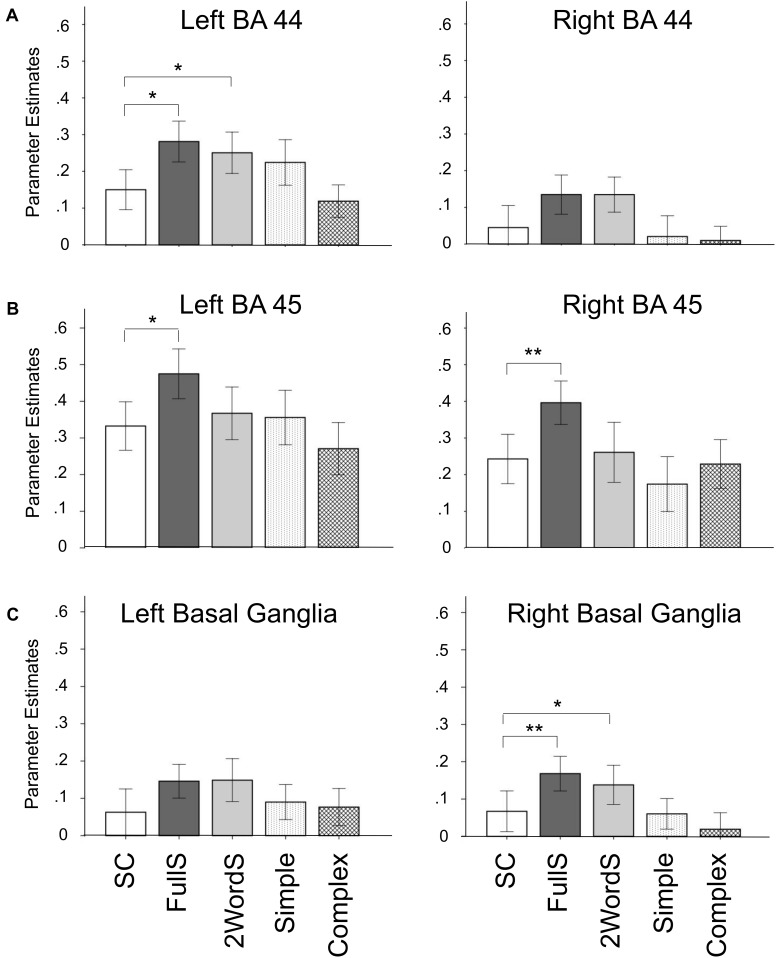
Reduced activation for Small Clause in anatomically defined syntax-related regions of interest. Parameter estimates per condition were extracted from 6 anatomically defined regions of interest and average group parameter estimates are shown by condition in bilateral BA 44 **(A)**, BA 45 **(B)**, and Basal Ganglia **(C)**. ^∗^*p* < 0.05; ^∗∗^*p* < 0.02; SC, Small Clauses; FullS, Full Sentences; 2WordS, Two-word Control Sentences; Simple, Simple Compounds; Complex, Complex Compounds.

In a complimentary analysis, parameter estimates for activation for SC, FullS, 2WordS, Simple Compounds, and Complex Compounds were extracted from 6 ROIs, *a priori* anatomically identified regions known to be involved in the processing of syntax: bilateral BA 44, BA 45, and basal ganglia. Figure 4 depicts the mean extracted values per each condition across participants to allow comparisons across conditions. Tests of differences between conditions were conducted in selected predefined comparisons, including estimating the differences (1) between SC, FullS, and 2WordS conditions, and (2) between Simple and Complex Compound conditions.

Thus, we first tested a three-way ANOVA comparing SC, FullS, and 2WordS conditions. We found significant difference in activation by condition in left BA 44 [*F*(2,26) = 4.50, *p* = 0.02], bilateral BA 45 [left: *F*(2,26) = 3.96, *p* = 0.03; right: *F*(2,26) = 4.60, *p* = 0.02], and right basal ganglia [*F*(2,26) = 4.38, *p* = 0.02]; see Figure 4. Follow-up planned comparisons showed that, in left BA44, activation in the SC condition was reduced compared to both FullS [*t*(13) = 2.69, *p* = 0.02] and 2WordS [*t*(13) = 2.72, *p* = 0.02] conditions. A different pattern of between-condition differences was identified in the left and right BA45, where activation in the SC condition was reduced compared to FullS condition [left: *t*(13) = 2.94, *p* = 0.01; right: *t*(13) = 3.22, *p* = 0.007] but not compared to 2WordS condition. In the right basal ganglia, a pattern similar to that found in Left BA 44 emerged, with activation in the SC condition reduced compared to both FullS [*t*(13) = 3.07, *p* = 0.009] and 2WordS [*t*(13) = 2.19, *p* = 0.048] conditions.

Within the 6 anatomically defined ROIs, we also tested for potential difference in activation for Simple versus Complex Compounds, but we did not find significant differences in any of the tested ROIs (*p*s > 0.05).

## Discussion

### The Role of Broca’s Area and the Basal Ganglia in Language Processing

Our finding of the differential involvement of BA 44 and the basal ganglia in the processing of FullS vs. SC is especially significant in light of the recent finding that BA 44–Basal Ganglia network is a syntactic processing network showing very strong neural interconnectivity (Ardila et al., 2016a), thought to have been subject to positive selection in the course of human evolution. The basal ganglia are highly interconnected to cortical regions, especially in the frontal lobes, including Broca’s, via parallel anatomically and functionally segregated “loops” (Draganski et al., 2008; Frey et al., 2008; Ford et al., 2013)^8^. In addition, there is experimental evidence using animal models for a language-basal-ganglia-gene pathway. When inserted in mice, the humanized *FOXP2* alleles affected their basal ganglia, increasing dendrite lengths and synaptic plasticity of the medium spiny neurons in the striatum (e.g., Enard et al., 2009).

Our results, as well as the results reported in Progovac et al. (2018), are compatible with the idea that recent genetic mutations, including in *FOXP2*, in the line of descent of humans, increased synaptic plasticity and neuronal connectivity of the human brain (e.g., Hillert, 2014; Dediu, 2015), particularly in the frontal-striatal network, enabling human capacity for more complex language. This is consistent with the view of language and brain co-evolution, i.e., the idea that brain evolution was, at least in part, driven by the selective pressures to use more complex abstract/layered syntax.

The involvement of *FOXP2* in language and frontal-striatal brain network was directly established by a discovery that individuals with a certain mutation in the gene suffered from a developmental impairment affecting speech and language, among other symptoms (Lai et al., 2001). In addition, Liégeois et al. (2003) showed that the affected individuals not only exhibited under-activation in the Broca’s area and its right homolog, but that both the caudate nucleus and putamen, the structures of basal ganglia, were sites of morphological abnormality.

#### Full Sentences vs. Small Clauses

As reported in Sections “Reduced Brain Activation for Small Clauses in Syntax-Related Regions” and “Reduced Activation for Small Clause in Anatomically Defined Syntax-Related ROI,” we found greater activation in several regions, including the left BA 44 and the right basal ganglia for FullS (i.e., sentences with the TP layer) relative to their proto-syntactic counterparts, i.e., SC lacking the TP layer. Activations in the left BA 44 and in the right basal ganglia were also higher in the Two-Word Control Condition (i.e., Full TP Sentences with the same number of words as the Small Clause Condition) compared to SC, indicating that increased activation in these regions was not merely due to the difference in the number of words per string, but instead likely to the presence of additional layers of syntactic structure. On the other hand, we found no differential activation for various syntactic conditions in BA 45 (left and right). These results provide some support for BA 44 being specialized for processing abstract hierarchical syntax more than BA 45, consistent with Hagoort and Indefrey (2014: 356) claim that BA 44 activation is “driven more strongly by syntactic rather than semantic demands.”

These results are consistent with our hypothesis that processing of modern abstract hierarchical syntax relies more on the syntax-specialized networks, which connect the Broca’s area to the basal ganglia. In contrast, processing ancestral proto-syntactic structures relies on this network substantially less.

#### Compounds

With respect to processing compounds, we did not find significant effects in the postulated syntactic network: BA 44, BA 45, and the basal ganglia. One potential reason for this may be that the syntactic distinction between the two compound types involves a lower-level, less abstract functional layer than the categories of TP and DP, which distinguish FullS from SC in English. While it has been proposed that -*er* compounds may involve a vP layer (e.g., Roeper, 1999), this is not universally accepted (see Progovac, 2015 for other possible types of analyses and references). Another possibility for the contrast between our results in the clausal versus the compound conditions is that unlike the compounds, the clause contrast in English involves not just one, but two abstract functional categories, TP and DP (Determiner Phrase). It is possible that a single functional projection is too subtle to lead to detectable results using our methodology. Another potential reason for the lack of differential activation in the syntactic areas between the two types of compounds was the lack of semantic matching between these two conditions, with the two types of items containing different content words (see Section “Stimuli”) and consequently enough noise to wash out significant effects.

Nevertheless, the compounds provided some noteworthy results. We identified several regions in which activation differed between these conditions. Specifically, higher activation for complex relative to simple compounds was found in the right precuneus, whereas higher activation for simple compared to complex compounds was found in a cluster spanning the inferior temporal lobe and fusiform gyrus (BA 37/19), and a second cluster spanning a portion of the cingulate cortex and superior medial frontal gyrus cortex (BA 24/6). BA 37 (an area located in the posterior portions of the fusiform gyrus and inferior temporal gyrus of the temporal lobe) has been implicated in both visual processing and semantic language processing, including tasks involving naming, concreteness, and metaphoricity (see a recent meta-analysis by Ardila et al., 2015). More specifically, BA 37 is the area where visual processing (e.g., drawing, face recognition) and certain non-compositional semantic processing (e.g., concreteness, metaphor) come together (*Brodmann’s Interactive Atlas;^9^* Bookheimer, 2002).

It would seem unsurprising that processing of simple compounds activated an area associated with concreteness and metaphoricity, given that these compounds typically consist of highly concrete pieces used metaphorically (e.g., *turn-coat, wag-tail, cry-baby*). What is interesting, however, is that our –*er* compounds, which also contained highly imageable pieces (e.g., *boot-licker, whistle-blower, heart-breaker*), did not show the same level of activation in these areas. The overlay of abstract syntactic structure that characterizes these latter compounds, in contrast to the fossil compounds, likely rendered their imageability less direct (i.e., less raw)^10^. Thus, our results suggest that the semantic dimension of concreteness-versus-abstractness, along with the structural (flat-versus-hierarchical) dimension, is relevant in human language evolution, with proto-syntax associated with the concreteness end, and modern hierarchical syntax with the abstractness end of the spectrum.

Another possible explanation for the increased activation in BA 37 with simple compounds is that the incomplete thematic nature of the simple compounds (the expression of only one argument) is registering as a semantic/thematic violation, thus activating semantics-related processing in BA 37. This would be in line with the report in Shetreet et al. (2010) that BA 37 may be partly responsible for reacting to the omission of optional arguments, as found in examples like “He ate.” However, their finding implicated the left BA 37, while we found an effect in the right BA 37. Finally, both imageability and thematic incompleteness may be contributing to the observed effect.

Interesting evidence in line with our findings comes from the investigations of a hereditary language disorder in the KE family (Fisher et al., 1998), due to a mutation in the *FOXP2* gene, as mentioned in the previous section. In an fMRI study, Liégeois et al. (2003) found that the affected family members showed a more posterior and more extensively bilateral pattern of activation, as well as under-activation in the Broca’s area and its right homolog, while the unaffected members exhibited a typical left-dominant activation involving frontal areas. It was suggested in Liégeois et al. (2003) that the overactivation of the areas outside of the language network reflected the recruitment of a compensatory circuit in response to dysfunction within the normal circuit. The KE family investigations represent an important example of “cross-pollination” between biological and language science fields.

## Conclusion and Future Prospects

The evolution-of-syntax framework affords unique and precise tools for dissecting linguistic structures for neuroimaging investigations, structures of various complexity levels (from syntax and proto-syntax). This approach is promising with respect to its potential to bring together the fields of theoretical linguistics, neuroscience, and genetics, providing a platform from which to consider how the gradual accretion of syntactic complexity influences the evolution of the brain, and vice versa. As such, it is well-positioned to shed new and specific light on the co-evolution of language and the brain.

Overall, our results provide some initial evidence for our prediction that the processing of proto-syntactic structures is supported less by the specialized syntactic brain networks, those that have been enhanced more recently in evolution, including the Broca’s–Basal Ganglia network. The bolstering of this network by abundance of neural connections has been suggested to be a recent evolutionary development, leaving open the possibility that the pressures to process ever more and more complex and abstract syntax contributed to this evolutionary path of the brain. These findings not only lead to new insights into the neuroscience of language, but they can also inform linguistic theorizing (e.g., by helping one choose between competing syntactic approaches to the proto-syntactic structures investigated here).

Moreover, cross-linguistic research along these lines promises to identify additional tools of this kind, as different languages make available syntactic structures and distinctions that are not there in English. As one example, Progovac et al. (2018) found that the basal ganglia showed increased activation with the processing of Serbian transitive sentences (instantiating a vP layer) in contrast to matched middle sentences (analyzed as lacking the vP layer). Straightforward contrasts like this are not available in English, as English does not have a grammaticalized category of middles, demonstrating how different languages provide different possibilities for testing. Finding both converging and diverging results across languages would be informative, as such results would point to universal as well as language-specific processing strategies, and possibly to different paths for the co-evolution of language and the brain.

## Ethics Statement

This study was carried out in accordance with the recommendations of ‘Wayne State University Institutional Review Board Committee’. All subjects gave written informed consent in accordance with the Declaration of Helsinki. The protocol was approved by the ‘Wayne State University Institutional Review Board Committee’.

## Author Contributions

LP has contributed the theoretical framework, as well as the linguistic data and analysis for the design of the fMRI experiments. NO has designed and overseen the experiments, as well as the extraction and calculation of the results. NR has contributed to the genetic background of the article, as well as to the theoretical framing of the discussion. WA and RL have recruited the participants and were directly involved in conducting the neuroimaging experiments. LT has been involved in the extraction and calculations of the results, and to some extent so were also WA and RL.

## Conflict of Interest Statement

The authors declare that the research was conducted in the absence of any commercial or financial relationships that could be construed as a potential conflict of interest.

## Footnotes

1 These approximations of proto-grammar show different properties compared to their more complex counterparts, including the lack of Move and recursion, as discussed in Progovac (2015, and references cited there). In this view, Subjacency effects (resistance to Move with some present-day constructions) are seen as epiphenomena of evolutionary tinkering.

2 The claim is that this type of two-slot mold was used in the proto-syntactic stage, rather than these specific compounds/words.

3 Clark et al. (1986) elicited novel -er compounds from young children. The youngest children consistently produced simple verb-noun compounds (‘grate-cheese,’ ‘rip-paper,’ and ‘bounce-ball’) instead of complex ones (‘cheese-grater,’ ‘paper-ripper,’ and ‘ball-bouncer.’) This suggests that children start with the simpler, more primary structure, before building more complex counterparts.

4 The –ed suffix on the main verbs in both (6) and (7) signals a participle form, typically associated with aspect and/or voice, rather than past tense. To appreciate this, consider that such –ed participles can occur even when the reference is to a future time (e.g., The problem will be solved.) Tense is marked only in (7), surfacing on the auxiliary verb. Also, DP in (7′) stands for Determiner Phrase, another abstract syntactic layer of structure built upon a Noun Phrase, the layer that would not have been available in the initial stages of language evolution. Even if (6) and (7) in English are analyzable as containing another layer of structure attributable to the participle –ed morpheme, the examples in (6) are still measurably flatter/simpler than the counterparts in (7), lacking the TP and DP layers.

5 Broca’s aphasics have been reported to have difficulties comprehending structures involving syntactic movement (e.g., Caramazza and Zurif, 1976; Zurif, 1995; Grodzinsky, 2000). However, it has also been reported that there is a much more complex and indirect relationship between the damage to the Broca’s area and syntactic processing deficits (e.g., Mohr et al., 1978; Novick et al., 2010; Thothathiri et al., 2012).

6 Although we did not find an effect in Broca’s area, transitives, compared to middles, evoked greater activation in the precentral gyrus (BA 6), proposed to be part of the “Broca’s complex” (e.g., Ardila et al., 2016b).

7 To overcome this limitation, one can force the same vocabulary on both conditions, such as (turn-coat/coat-turner; pick-pocket/pocket-picker; scare-crow/crow-scarer). However, this would result in unnatural and infrequent (never-heard-before) compounds, which would raise problems of different nature.

8 That the striatum is involved in syntactic processing was shown in lesion studies (e.g., Moro et al., 2001; Teichmann et al., 2005, 2008; Newman et al., 2010).

9 http://www.fmriconsulting.com/brodmann/BA37.html

10 Simple verb-noun compounds are known for their highly visceral effect, and for their specialization for insult (Progovac and Locke, 2009: 344). In medieval times, these compounds sometimes showed unquotable coarseness (Weekley, 1916) and were used for a very expressive way of naming, which flourished in 13th and 14th century, yielding thousands of tokens. Darmesteter (1934: 443) noted the “inexhaustible artistic beauty and richness” of verb-noun compounds in French. Mihajlović (1992) collected over 500 Serbian place and people names in the form of verb-noun compounds, reporting that these condensed compositions pack in them “frozen fairy tales, proverbs, and ancient wisdoms and metaphors”(1992: 8–9).

## APPENDIX: STIMULI USED IN THE DIFFERENT EXPERIMENTAL CONDITIONS^11^

### Small Clauses (SC)

Meeting adjourned.
Issue resolved.
Battle won.
Battle won.
Body found.
Obama elected.
Warning heeded.
Apology accepted.
Lesson learned.

Request approved.
Suspect arrested.
Hearing postponed.
Message received.
Fingers crossed.
Fingers crossed.
Crisis averted.
Permission granted.
Point taken.

Case closed.
Mission accomplished.
Innocence lost.
Help wanted.
Class canceled.
Class canceled.
Signature needed.
Problem solved.
Dishes done.

### Full Sentences (FullS)

The problem is solved.
The case is closed.
The point is taken.
The class is canceled.
The hearing was postponed.
Obama was elected.
Obama was elected.
My fingers are crossed.
The warning was heeded.

The request is approved.
The battle is won.
The mission was accomplished.
Innocence was lost.
The suspect was arrested.
The meeting is adjourned.
The issue was resolved.
The issue was resolved.
The message is received.

A body was found.
A signature is needed.
The dishes are done.
The crisis was averted.
The apology is accepted.
The lesson was learned.
Help is wanted.
The permission is granted.
The permission is granted.

### Two Word Control Sentences (2WordS)

Help arrived.
Help arrived.
Snow melted.
Change followed.
Mistakes happen.
Stars shine.
Tears dropped.
Fires spread.
Grass grew.

Cracks developed.
Errors emerged.
Errors emerged.
Love stinks.
Fruit ripened.
Flowers bloomed.
Spring arrived.
Disruption occurred.
Morning arrived.

Fog lifted.
Balls bounced.
Confusion ensued.
Clouds gathered.
Night fell.
Lies hurt.
Lies hurt.
Life happened.
Cheers erupted.

### Simple Compounds

play-boy
scape-goat
cry-baby
saw-bones
scatter-brain
kill-joy
fall-guy
fall-guy
dip-stick

pick-pocket
worry-wart
cut-purse
busy-body
tattle-tale
skin-flint
blabber-mouth
blabber-mouth
spit-fire

spoil-sport
hunch-back
turn-coat
turn-coat
tell-tale
scoff-law
dare-devil
lack-wit
copy-cat

### Complex Compounds

tax-payer
problem-solver
boot-licker
brick-layer
brick-layer
trouble-maker
mind-reader
heart-breaker
meat-eater

risk-taker
whistle-blower
woman-hater
bird-watcher
match-maker
party-pooper
party-pooper
rule-breaker
head-turner

bone-crusher
ball-breaker
dress-maker
man-eater
man-eater
truck-driver
story-teller
grave-digger
truth-seeker
